# Effect of Sweeteners on the Solvent Transport Behaviour of Mechanically-Constrained Agarose Gels

**DOI:** 10.3390/gels4010023

**Published:** 2018-03-16

**Authors:** Isamu Kaneda

**Affiliations:** Food Physical Chemistry lab, Rakuno Gakuen University, Ebetsu, Hokkaido 069-8501, Japan; kaneda-i@rakuno.ac.jp; Tel.: +81-11-388-4701

**Keywords:** agarose gel, compression, solvent transport, sucrose, xylitol

## Abstract

Investigating the solvent transport behaviour of edible gels is important because it is strongly related to flavour release. We previously reported the solvent transport behaviour of mechanically-constrained agarose gels. These studies clearly showed that agarose gels can be treated as soft porous bodies. Herein, we investigated the effect of sweeteners on the solvent transport speed, which is an important issue in the food industry, using sucrose and xylitol. Sucrose caused a concentration-dependent reduction in solvent transport speed. One of the reasons for the effect is that the solvent to which sucrose was added reduced solvent flow speed within the porous agarose network. This finding provides valuable information for flavour release from compressed gels. Moreover, we found a similar effect for xylitol, which is a promising candidate for substituting sucrose in low-calorie foods. This study would provide basic knowledge for the development of a new type of low-calorie foods.

## 1. Introduction

Agarose is a neutral polysaccharide derived from Rhodophyta with a structure consisting of repeating 1,3-binding β-d-galactose and 1,4-binding 3,6-anhydro-α-l-galactose units. Agarose is insoluble in water at low temperatures owing to its double helix structure [1], but dissolves at high temperatures due to dissociation of the double helix. As agarose dissolved in water at high temperatures is allowed to cool and the thermal motion of the polymer chain is restrained, hydrogen bonds reform to create the double helix structure. If the polymer concentration is high enough, the helical structure forms a three-dimensional network [2]. As the helical structure is not water soluble, the 3D network structure does not swell in water. Therefore, a large quantity of water is held within the network structure, affording an agarose gel.

We have investigated the solvent transport behaviour of constrained agarose gel as a model system for studying flavour release behavior [3,4]. Compression load occurs when the agarose gel is compressed, but the load is relaxed. We found that the volume decreased with time by approximately the same amount as the compression load relaxation time when the change in gel volume was observed simultaneously. This phenomenon can be successfully explained using stress-diffusion coupling theory [5], which shows that agarose gels can be treated as soft porous bodies.

As most food flavourings are water soluble, solvent transport behaviour and flavour release are expected to be closely related. Agarose is often applied as a gelling agent in sweet desserts. Therefore, changes in the physical properties of agarose gel with the addition of saccharide (sweetener) are an important research theme in food processing. The mechanical and thermal properties of agarose reportedly improve with sucrose addition [6,7]. Furthermore, the syneresis often observed in gelatinous food containing agarose is controlled by sucrose addition [8]. A comprehensive review of sucrose release behaviour from agarose gels has been published [9]. However, the most of these studies are on the passive diffusion behaviour of sucrose in agarose gels. We can investigate the squeeze-out speed of the solvent from the compressed agarose gels using our unique experimental system [3]. It is worth considering flavour release during the eating action.

## 2. Results and Discussion

### 2.1. Relationship between Compression Load Relaxation and Volume Change

**Figure 1:**
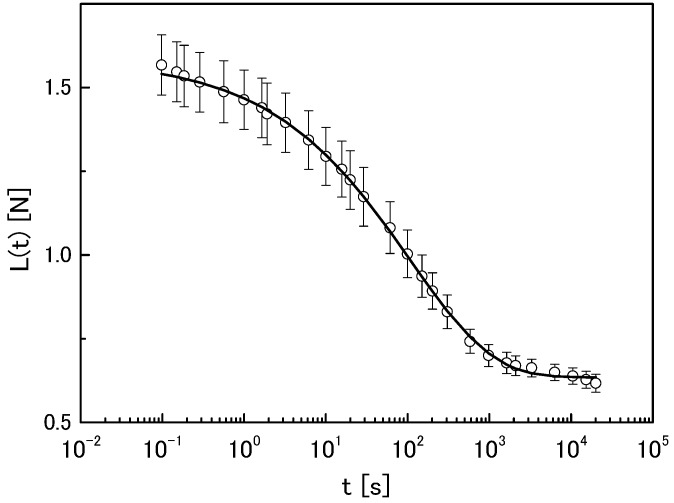
Time profile of compression load for 1.5-wt % agarose gel. The line represents the best fit result of analysis using Equation (1).

The time profile of the change in the compression load of agarose gel (1.5 wt %) is shown in Figure 1. The compression load decreased exponentially with time, but did not relax completely, reaching a plateau after 10^4^ s. We analysed this relaxation behaviour using a stretched exponential function (Equation (1)):

(1) $$L\left(t\right)=L_{0}\exp\left({\left(-\frac{t}{\tau_{M}}\right)}^{a}\right)+L_{r}$$

where *L*_0_ is the relaxed component of the compression load, *τ_M_* is the relaxation time, *L_r_* is the residue of the compression load, and *a* is the stretched index. The solid line in Figure 1 represents the fitting result using Equation (1) (the actual values of the parameters are listed in Table 1 as “control”). The experimental data fitted well with Equation (1), indicating that the compression load of agarose gels relaxed over time, but not completely. 

**Figure 2:**
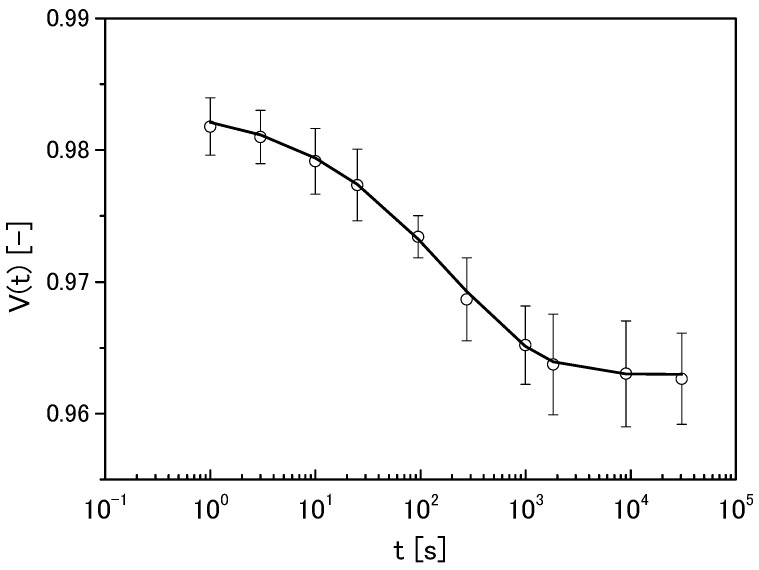
Time profile of the volume change in 1.5-wt % agarose gel. The line represents the best fit result of analysis using Equation (2).

The time profile of the volume change in agarose gel (1.5 wt %) is shown in Figure 2. The solid line in Figure 2 is the result of analysis using Equation (2) (the actual values of the parameters are listed in Table 2 as “control”)

(2) $$V\left(t\right)=V_{0}\exp\left({\left(-\frac{t}{\tau_{V}}\right)}^{b}\right)+V_{r}$$

where *V*_0_ is the reduced component of the volume change ration, *τ_V_* is the time constant of the volume change speed, *V_r_* is the residue of the volume change ratio, and *b* is the stretched index. Comparing Figure 1 with Figure 2 showed that this decreasing behaviour was synchronous. This was not coincidental, but attributed to a correlation between these two properties. As we have reported previously [3,4], this correlation can be explained using the stress-diffusion coupling theory [5]. By assuming that agarose gels are soft porous bodies, solvent in the capillary would be squeezed out by compression. Therefore, the speed of this solvent removal from the constrained gels was estimated from the mechanical relaxation. This was valuable because measuring the compression load was easier than measuring the solvent transport speed.

### 2.2. Effect of Sucrose and Xylitol on Solvent Transport from Constrained Agarose Gels

To determine the effect of sucrose and xylitol on the solvent transport speed, agarose gels (1.5 wt %) were prepared with solvent containing sucrose or xylitol (10–50 wt %), denoted herein as S-Y and X-Y, respectively, where Y indicates the amount of sucrose or xylitol in the solvent (in wt %).

**Figure 3:**
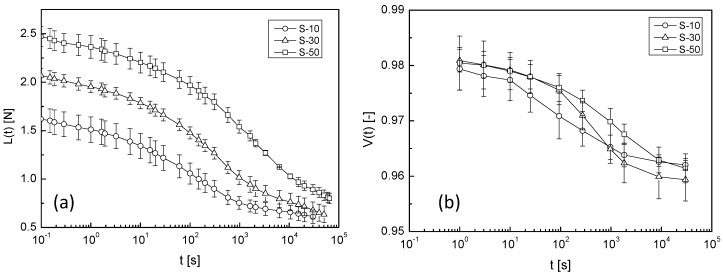
Time profile of (**a**) compression load and (**b**) volume change in samples containing sucrose. Circles, triangles, and squares denote S-10, S-30, and S-50 samples, respectively. Error bars represent the standard deviation. The lines are an eye-guide.

The compression load change and volume change in samples containing sucrose are shown in Figure 3a,b, respectively. The compression load increased with increasing sucrose concentration, as clearly shown in Figure 3a. Furthermore, the relaxation time appeared to increase with increasing sucrose concentration.

**Figure 4:**
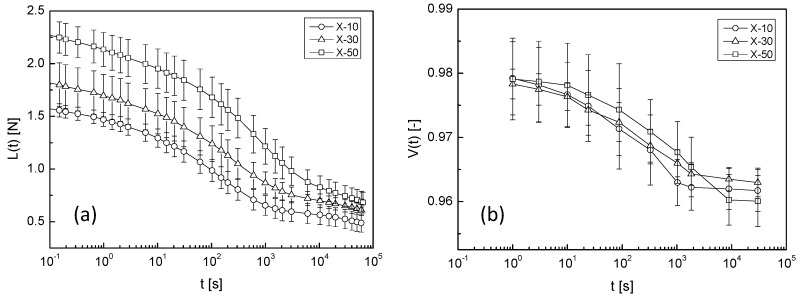
Time profile of (**a**) compression load and (**b**) volume change in samples containing xylitol. Circles, triangles, and squares denote X-10, X-30, and X-50 samples, respectively. Error bars represent the standard deviation. The lines are an eye-guide.

The results obtained for samples containing xylitol are shown in Figure 4. Xylitol addition had similar effects on *L*(*t*) and *V*(*t*) to sucrose addition, namely, increased *L*(*t*) and increased time taken for the volume change with increasing sweetener concentration. 

For a quantitative evaluation of the effect of sucrose and xylitol, the experimental curves (*L*(*t*) and *V*(*t*)) were analysed using Equations (1) and (2) for the entire samples. The results are listed in Table 1 and Table 2.

**Figure 5:**
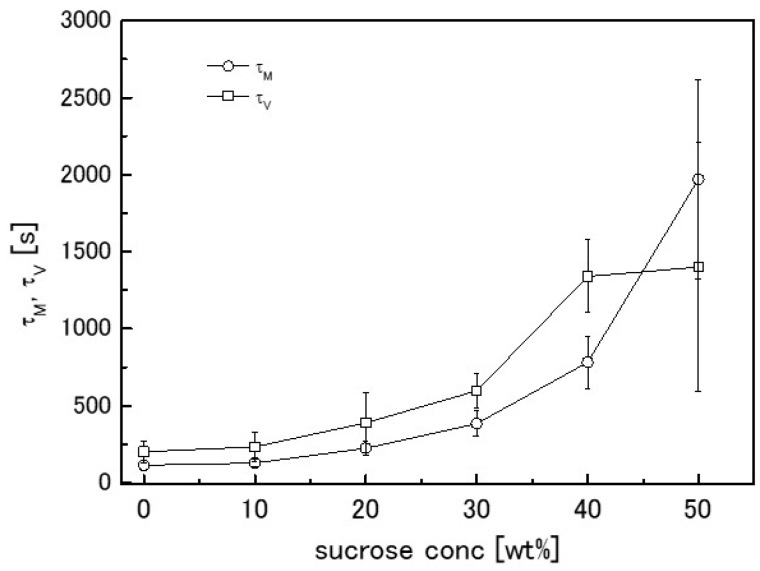
Dependence of time constants *τ_M_* (circles) and *τ_V_* (squares) on sucrose concentration. Error bars represent the standard deviation.

**Figure 6:**
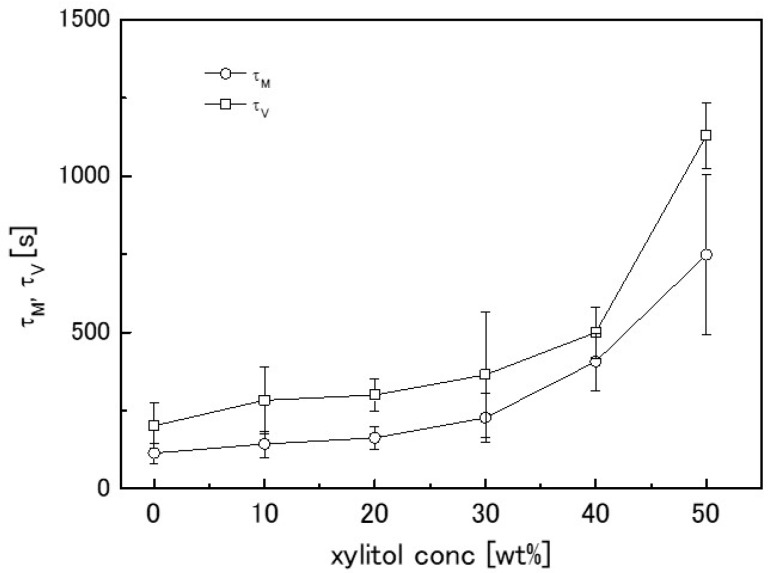
Dependence of time constants *τ_M_* (circles) and *τ_V_* (squares) on xylitol concentration. Error bars represent the standard deviation.

To easily check the effect of adding sweeteners on the solvent transport behaviour, the mechanical relaxation time (*τ_M_*) and the time constant (*τ_V_*) of the volume change of the agarose gel containing the sweeteners are plotted against their concentrations. The effect of sucrose and xylitol addition are shown in Figure 5 and Figure 6, respectively. Sucrose and xylitol were found to effectively reduce the speed of solvent transport from the mechanically-constrained gel. However the solvent transport speed reducing effect of xylitol is less than sucrose. A possible explanation for this phenomena provided below (in Section 2.4).

### 2.3. Influence of Sweetener Addition on Network Structure of Agarose Gel

**Figure 7:**
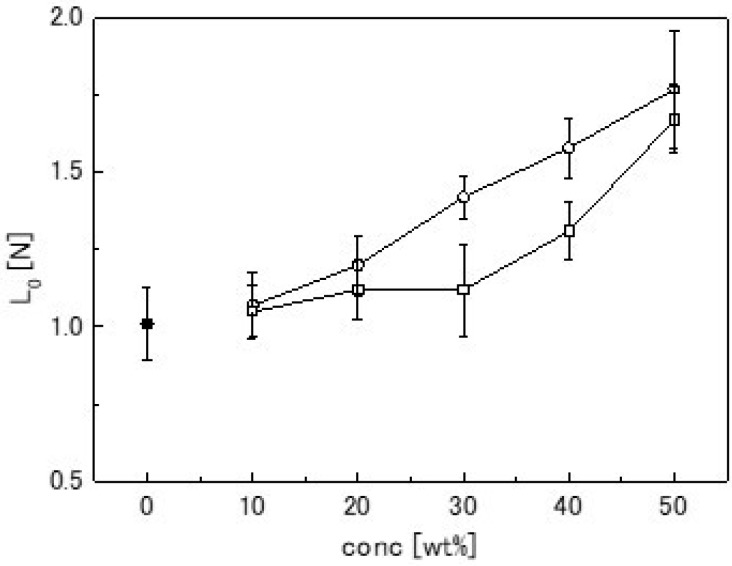
Gel strength (*L*_0_ in Equation (1)) of samples containing various sweetener concentrations. Closed circles, open circles, and open squares denote the control (1.5-wt % agarose), sucrose series, and xylitol series, respectively.

The network structure of agarose gel has been reported to change with sucrose addition [6,7,8]. The enthalpy of gelation and Young’s modulus reportedly increased with sucrose addition. In this study, the compression load also increased with sucrose addition, as shown in Figure 3a. In contrast, the influence of xylitol on the Young’s modulus of agarose gel has rarely been reported. Xylitol was also clearly effective in increasing the Young’s modulus of agarose gel, as shown in Figure 4a. *L*_0_ in Equation (1) represents the relaxed component of the compression load. The sweetener concentration-dependency of *L*_0_ is shown in Figure 7. *L*_0_ clearly increased with increasing sweetener concentration. This result suggested that a change occurred in the network structure formed by agarose gel with sucrose or xylitol addition. Therefore, freeze-dried gel samples were observed using SEM.

**Figure 8:**
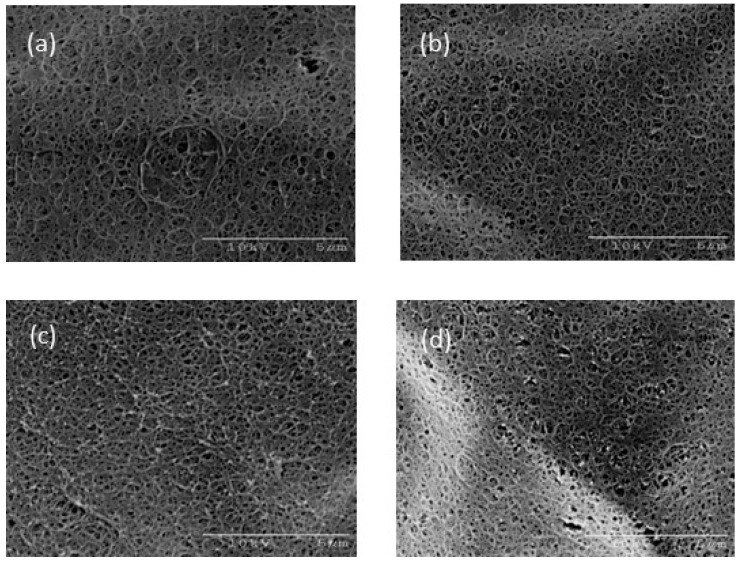
SEM images of (**a**) S-10, (**b**) S-20, (**c**) S-30, and (**d**) S-50. The scale bars indicate 5 μm.

**Figure 9:**
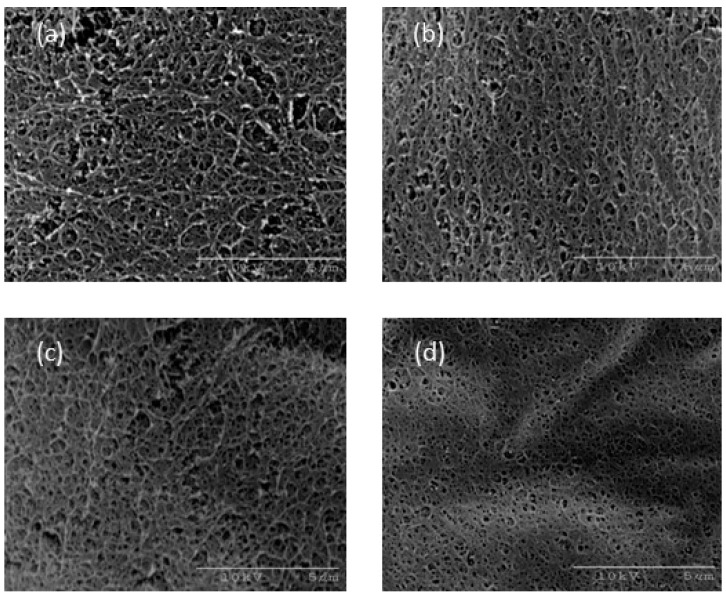
SEM images of (**a**) X-10, (**b**) X-20, (**c**) X-30, and (**d**) X-50. The scale bars indicate 5 μm.

Scanning Electro-Microscopy (SEM) images of the agarose gel structure with sucrose addition are shown in Figure 8. As the sucrose concentration increased, the mesh size of the agarose gel network seemed to become smaller. The results for xylitol addition using a similar method are shown in Figure 9. A decrease in the mesh size of the agarose gel network was observed with xylitol addition, as observed for sucrose addition above. Agarose molecules, with their spiral structure, are known to associate to form a gel network structure. The sequence state of the spiral structure has been reported to change with sucrose addition, resulting in the strength of the gel network structure changing. This phenomenon was thought to cause the increasing Young’s modulus observed in this study. Xylitol also clearly had a similar effect on the agarose gels in this study.

### 2.4. Relationship between Structural Changes and Solvent Transport Behaviour of Agarose Gel Networks

The syneresis of agarose gel is thought to be empirically controlled by sucrose addition. However, the mechanism of this phenomenon on the molecular level remains unclear. The improvements in the mechanical characteristics resulting from sucrose addition discussed in Section 2.3 are partly due to changes in the gel network. The moving speed of solvent molecules is thought to decrease intuitively owing to the mesh size of the gel network decreasing. However, the mesh size of the gel network, as observed by SEM (Figure 8 and Figure 9), was too large to prevent solvent transport. Furthermore, their size was estimated to range from 10 to 100 nm [10,11,12].

As mentioned above, the solvent transport behaviour of mechanically-constrained agarose gel can be explained using the stress-diffusion coupling theory, which treats agarose gels as soft porous bodies. Considering the agarose gels to be soft porous bodies, an increase in inner pressure would occur during compression, which should squeeze solvent out of capillaries in the gel. The flow quantity (*Q*) can be estimated using the Hagen–Poiseuille law (Equation (3)):

(3) $$Q\propto \frac{r^{4}}{\eta L}P$$

where *P* is the increase in pressure, *r* is the capillary radius, *L* is the capillary length, and *η* is the flow liquid viscosity. The solvent viscosity was the factor determining the flow quantity if the shape of the capillary and pressure increase were constant. According to the literature, sucrose–water solutions have viscosities of 1.06 mPa s (10 wt %) and 10.0 mPa s (50 wt %) at 30 °C. Using these viscosity values and Equation (3), the flow quantity of S-10 was estimated to be ten-fold that of S50. In contrast, the value of *τ_M_* for S-10 was 15-fold higher than that of S-50, with the *τ_V_* for S-10 six-fold higher than that of S-10 (see Table 1). These values were similar to the estimated value mentioned above. Therefore, the reduction in solvent transport speed resulting from sucrose addition might be mainly due to the rise in solvent viscosity. The effect of xylitol was expected to be similar to that of sucrose. As viscosity data for xylitol–water solutions were not available in the literature, the viscosities were measured, giving approximately 1 mPa s and 6 mPa s for 10-wt % and 50-wt % solutions, respectively. The viscosity thickening effect of xylitol was slightly inferior to that of sucrose, but in the same order of magnitude, and the increase in *τ_M_* and *τ_V_* was thought to be dependent on the solute levels, with similar dependencies observed in both the xylitol and sucrose systems. Water molecules reportedly strongly hydrate sugar molecules in agarose, water, and sugar-containing systems, resulting in their removal from the vicinity of the agarose molecules. Therefore, sucrose and xylitol may become concentrated in the low density phase of the agarose gel, namely in capillary of soft porous body. If the sweetener aqueous solution is concentrated locally, the viscosity of the solvent rises, and is thought to be observed as a drop in solvent transport speed from the mechanically-constrained agarose gel macroscopically.

## 3. Conclusions

The effect of sweetener on the speed of solvent transport speed from mechanically-constrained agarose gels was investigated. Empirically, it was known that sucrose prevents syneresis from agarose gels, but the mechanism of this phenomenon remained unclear. Herein, we estimated the solvent transport speed from constrained agarose gels containing sweeteners using a unique experimental system. Both sucrose and xylitol caused a concentration-dependent reduction in the solvent transport speed. This phenomenon is attributed to two factors: (i) Changes in the microscopic structure of the agarose gels caused by adding sweeteners, and (ii) changes in the viscosity of the solvent containing sweeteners. We showed that agarose gels can be treated as soft porous bodies in a previous study. As the viscosity of the solvent would increase with the addition of sweetener, it is expected that the flow quality (speed) would decrease according to the Hagen–Poiseuille law.

## 4. Materials and Methods

### 4.1. Materials

Agarose type-IV (Sigma-Aldrich, St. Louis, MO, USA) containing less than 0.25% sulfate was purchased and used without further purification. For all samples, the agarose concentration was 1.5 wt %. Sucrose and xylitol aqueous solution were used as solvents for the gels. Their concentrations were ranging from 0 to 50 wt % in distilled water. Sample gels were prepared as follows; Agarose powder was dispersed in the solvent and stirring at room temperature for 18 h. The dispersion was heated at 95 °C for 1 h to completely dissolve. The hot solution was poured into a plastic tube (φ = 20 mm) and both ends of the tube were sealed. The tube was then immediately placed in a water bath for temperature control. After 24 h of quenching in the water bath at 10 °C, the sample gels were cut to a length of about 20 mm using a razor blade. The cylindrical gels were immersed in solvent, which was used in the preparation of gels, and incubated at 5 °C for at least five days to reach an equilibrated state before the experiments. The diameter and length of gel samples were measured with a caliper immediately before experiments. 

### 4.2. Monitoring of Compression Load and Volume Change 

The measurement system has been described elsewhere [3]. Compression and monitoring of the compression load were performed using an INSTRON MINI 55 instrument (Instron, Norwood, MA, USA). To cancel the surface tension at the surface of gel samples and prevent drying, the gel samples were immersed in their solvent during experiment and kept their temperature at 30 °C. The sample gel was compressed at a rate of 1 mm/s and, when the compression strain reached 0.05, the compression was kept constant. Changes in the compression load were monitored for 18 h. The pictures of the side view of the sample was taken using a charge-coupled device (CCD) camera. The top and bottom surfaces of the cylindrical gel were sealed with cyanoacrylate to prevent slipping. As the gel deformed to a barrel-like shape, the width was measured at five different points and the average width (*w_m_*) was obtained. The height of the gel was determined during compression load measurements and the gel volume (*v*) was calculated using Equation (4), with each measurement performed at least in triplicate. Digital images of the samples were analysed using image analysis software (Image-J (NIH), Bethesda, MD, USA).

(4) $$v=\pi {\left(\frac{w_{m}}{2}\right)}^{2}\cdot h$$

### 4.3. Viscosity Measurements

The apparent viscosities of the solvents were measured by a rotation rheometer (ARES: TA instruments, New Castle, DE, USA) equipped with a bob (diameter, 16 mm) and cup (diameter, 16.5 mm). The steady state viscosity at 50 s^−1^ was measured at 30 °C. 

### 4.4. Scanning Electron Microscopy (SEM) Observation

The microstructure of the agarose gels was observed using a scanning electron microscope (HITACHI S-2460N, Tokyo, Japan). The sample gels were immersed in distilled water for at least 10 days to remove sucrose or xylitol. The washed gels were rapidly cooled in liquid nitrogen, and then the completely freeze-dried samples were cleaved to expose the torn surfaces and coated with Pt-C on the SEM sample table.

## Figures and Tables

**Table 1:** Characteristic parameters of compression load relaxation analysed using Equation (1).

Samples		*L*_0_ (N)	*L*_r_ (N)	*τ_M_* (s)	*a* (-)
control		1.01 ± 0.119	0.602 ± 0.0572	114 ± 33.1	0.412 ± 0.0220
Sucrose	S-10	1.07 ± 0.108	0.627 ± 0.0752	128.9 ± 26.5	0.355 ± 0.0552
	S-20	1.20 ± 0.0969	0.708 ± 0.0663	2235 ± 46.9	0.377 ± 0.0311
	S-30	1.42 ± 0.0694	0.679 ± 0.100	385 ± 80.7	0.390 ± 0.0378
	S-40	1.58 ± 0.0970	0.695 ± 0.0475	782 ± 170	0.362 ± 0.0302
	S-50	1.77 ± 0.191	0.765 ± 0.141	1970 ± 648	0.367 ± 0.0610
Xylitol	X-10	1.05 ± 0.0832	0.559 ± 0.107	143 ± 41.9	0.422 ± 0.0187
	X-20	1.12 ± 0.0961	0.620 ± 0.0260	163 ± 36.6	0.361 ± 0.0353
	X-30	1.12 ± 0.149	0.669 ± 0.0591	227 ± 78.7	0.360 ± 0.0469
	X-40	1.31 ± 0.0937	0.795 ± 0.0843	407 ± 91.6	0.388 ± 0.0296
	X-50	1.67 ± 0.105	0.680 ± 0.105	749 ± 257	0.340 ± 0.0386

**Table 2:** Characteristic parameters of volume change analysed using Equation (2).

Samples		*V*_0_ × 10^2^ (-)	*V_r_* × 10^2^ (-)	*τ_V_* (s)	*b* (-)
control		2.06 ± 0.221	95.5 ± 1.70	202 ± 72.0	0.514 ± 0.0819
Sucrose	S-10	2.07 ± 0.144	96.2 ± 0.131	233 ± 96.4	0.423 ± 0.0255
	S-20	2.13 ± 0.348	96.0 ± 0. 273	390 ± 191	0.518 ± 0.142
	S-30	2.15 ± 0.309	96.0 ± 0. 403	597 ± 109	0.629 ± 0.0649
	S-40	1.95 ± 0. 201	95.9 ± 0. 264	1340 ± 236	0.538 ± 0.0481
	S-50	1.69 ± 0.579	96.1 ± 0. 300	1400 ± 806	0.708 ± 0.131
Xylitol	X-10	1.97 ± 0.0671	95.8 ± 0.600	283 ± 108	0.625 ± 0.159
	X-20	1.76 ± 0.111	95.8 ± 0. 241	300 ± 52.1	0.592 ± 0.0583
	X-30	1.60 ± 0.149	95.7 ± 0. 248	365 ± 201	0.565 ± 0.144
	X-40	1.93 ± 0.135	96.2 ± 0. 287	500 ± 82.6	0.552 ± 0.0624
	X-50	1.98 ± 0. 237	96.0 ± 0. 410	1130 ± 104	0.507 ± 0.0500
